# Species differences in ocular pharmacokinetics and pharmacological activities of regorafenib and pazopanib eye‐drops among rats, rabbits and monkeys

**DOI:** 10.1002/prp2.545

**Published:** 2019-11-20

**Authors:** Shinya Horita, Miwa Watanabe, Mai Katagiri, Hiroaki Nakamura, Hiroki Haniuda, Tomoyuki Nakazato, Yoshiyuki Kagawa

**Affiliations:** ^1^ R&D Division Kyowa Kirin Co., Ltd. Sunto‐gun Shizuoka Japan; ^2^ Department of Clinical Pharmaceutics School of Pharmaceutical Sciences University of Shizuoka Sunto‐gun Shizuoka Japan; ^3^ Production Division Kyowa Kirin Co., Ltd. Sunto‐gun Shizuoka Japan

**Keywords:** age-related macular degeneration, eye‐drop, ocular pharmacokinetics and pharmacological activities, pazopanib, regorafenib, species differences, vascular endothelial growth factor

## Abstract

Age‐related macular degeneration (AMD) is the leading cause of severe vision impairment in patients over the age of 60 years. Choroidal neovascularization (CNV) is the hallmark of neovascular AMD and vascular endothelial growth factor (VEGF) plays a causal role in the formation of CNV. Although regorafenib and pazopanib, small molecule VEGF receptor (VEGFR) inhibitors, were developed as eye‐drops, their efficacies were insufficient in clinical. In this study, we evaluated ocular pharmacokinetics and pharmacological activities of regorafenib and pazopanib after ocular instillation in multiple animal species. In rats, both regorafenib and pazopanib showed high enough concentrations in the posterior eye tissues to inhibit VEGFR. In laser‐induced rat CNV model, regorafenib showed clear reduction in CNV area. On the other hand, the concentrations of regorafenib and pazopanib in the posterior eye tissues were much lower after ocular instillation in rabbits and monkeys compared to those in rats. Pazopanib did not show any improvement in monkey model. Regorafenib was nano‐crystalized to improve its drug delivery to the posterior eye tissues. The nano‐crystalized formulation of regorafenib showed higher concentrations in the posterior segments in rabbits compared to its microcrystal suspension. From these studies, large interspecies differences were found in ocular delivery to the posterior segments after ocular instillation. Such large interspecies difference could be the reason for the insufficient efficacies of regorafenib and pazopanib in clinical studies. Nano‐crystallization was suggested to be one of the effective ways to overcome this issue.

**Abbreviations**
AMD: Age‐related macular degeneration
CNV: Choroidal neovascularization
VEGF: Vascular endothelial growth factor
VEGFR: VEGF receptor

## 1 INTRODUCTION

Age‐related macular degeneration (AMD) is a chronic disease in the posterior eye segment and the leading cause of severe vision impairment and legal blindness in patients over 65 years old in Western populations.^1^, ^2^, ^3^, ^4^ It has been estimated that the number of patients with AMD is going to increase in the next few decades because 25% of Asian people are going to be over 60 years old by 2050.^5^ AMD is classified into two subgroups, the non‐neovascular (nonexudative or dry) form and the neovascular (exudative or wet) form of the disease.^6^ Choroidal neovascularization (CNV) is the hallmark of neovascular AMD and causes leaking fluid, lipids and blood, and those leads to fibrous scarring.^3^ Vascular endothelial growth factor (VEGF) is a regulator of neovascularization and plays a causal role in the formation of CNV.^7^, ^8^ Anti‐VEGF therapy has remarkably improved visual outcomes in neovascular AMD patients, and ranibizumab and aflibercept are mainly used as anti‐VEGF agents. Intravitreal injections of ranibizumab and aflibercept not only prevent vision loss but also lead to significant visual gain.^9^, ^10^, ^11^, ^12^, ^13^, ^14^ However, the current anti‐VEGF therapy has multiple issues: invasive and frequent injections, high financial costs, and risk of ocular and systemic adverse events.^14^, ^15^, ^16^

To overcome these problems, small molecule inhibitors of VEGF receptors (VEGFR) have been developed as eye‐drop formulation.^6^ Regorafenib and pazopanib are multiple tyrosine kinase inhibitors which mainly targets VEGFRs. Regorafenib has been approved for the treatment of metastatic colorectal cancer, metastatic gastrointestinal stromal tumor and hepatocellular carcinoma, and pazopanib for advanced renal cell carcinoma and advanced soft tissue sarcoma.^17^, ^18^ Eye‐drop formulations of regorafenib (ophthalmic suspensions) and pazopanib (ophthalmic solutions) had been clinically developed for the treatment of neovascular AMD. However, the clinical development was terminated because of the lack of efficacy.[19, 20, 21] The eye‐drop formulations of regorafenib and pazopanib showed significant improvements in nonclinical laser‐induced CNV models in our study or another study,^22^ however, reasons for their poor efficacies in humans are still unclear.

Nano‐crystalization of drugs has been studied for the purpose of improving ocular drug delivery. Compared to conventional micro‐sized crystals, nano‐crystalization increase the surface area of particles which lead to increased dissolution rate and apparent solubility of drugs. In addition, nano‐crystals are beneficial for long time retainment on eye surface. Nano‐crystals can be retained in the cul‐de‐sac and its large surface area allows long‐term adhesion to the eye surface.^23^, ^24^, ^25^, ^26^

In this study, in order to consider the discrepancy observed in the efficacies between animal models and clinical studies, interspecies difference in ocular pharmacokinetics was investigated for regorafenib and pazopanib after ocular instillation. From the results, large interspecies difference was found in the ocular delivery to the posterior segments. The concentrations of regorafenib and pazopanib in the posterior segments were high in rats but low in rabbits and monkeys. The impact of nano‐crystalization was also investigated for regorafenib. The concentrations of regorafenib in the posterior eye segments were increased by nano‐crystalization. Our results indicated the importance to consider interspecies difference in ocular delivery to the posterior segments. Our results also suggested that nano‐crystalization is an effective way to increase ocular delivery in animal species where poor ocular delivery was observed with conventional microcrystal suspension.

## 2 MATERIALS AND METHODS

### 2.1 Materials

Regorafenib, 4‐[4‐ ({[4‐Chloro‐3‐(trifluoromethyl) phenyl]carbamoyl}amino)‐3‐fluorophenoxy]‐N‐methylpyridine‐2‐carboxamide monohydrate, was purchased from Selleck Chemicals Co., Ltd. and Active Biochem, Ltd.. Pazopanib, 5‐[[4‐[(2,3‐dimethylindazol‐6‐yl)‐methylamino]pyrimidin‐2‐yl]amino]‐2‐methylbenzenesulfonamide hydrochloride was purchased from SYNKinase Co., Ltd. Hydroxypropyl cellulose, sodium dihydrogen phosphate, sodium chloride and sodium hydroxide were purchased from FUJIFILM Wako Pure Chemical Corporation (Osaka, Japan). Light liquid paraffin and benzalkonium chloride were purchased from NACALAI TESQUE, Inc (Kyoto, Japan). Polysorbate 80 and D‐mannitol were purchased from Junsei Chemical Co., Ltd. Captisol was purchased from Ligand Pharmaceuticals, Inc. Aflibercept (40 mg/mL EYLEA^®^ Injection For Intravitreal Injection) was purchased from Bayer Yakuhin, Ltd. Mydrin‐P ophthalmic Solution was purchased from Santen Pharmaceutical Co., Ltd.. Scopisol solution was purchased from Senju Pharmaceutical Co., Ltd. (Osaka, Japan). Fluorescite Intravenous Injection 500 mg was purchased from Alcon Japan Ltd. (Tokyo, Japan).

### 2.2 Animals

All the animal tests in this study were conducted in accordance with the Guide for the Care and Use of Laboratory Animals. All the experimental protocols used in this study were approved by the Committee for Animal Experiments at Kyowa Kirin Co., Ltd., Shin Nippon Biomedical Laboratories, Ltd., Bozo Research Center Inc or Life Science Laboratories, Ltd.

In pharmacokinetic studies, male BN/CrlCrlj rats obtained from Charles River Laboratories Japan Inc, female Kbl:Dutch rabbits obtained from Kitayama Labes Co Ltd. and male cynomolgus monkeys obtained from Bozo Research Center Inc and Shin Nippon Biomedical Laboratories, Ltd. were used. In pharmacological studies, male BN/SsN Slc rats obtained from Japan SLC Inc and male cynomolgus monkeys obtained from Shin Nippon Biomedical Laboratories, Ltd. were used.

### 2.3 Regorafenib eye‐drops and Pazopanib eye‐drops

Regorafenib bulk powder and light liquid paraffin as dispersion medium were transferred into a vessel mixer of ARE‐310 (THINKY CORPORATION) with stainless milling balls. The pulverization was conducted to obtain regorafenib ophthalmic suspensions (regorafenib eye‐drops) at concentrations of 17.1‐24.1 mg/mL with micro‐size particles (average particle size: 4.62‐6.40 μm).

Regorafenib bulk powder and dispersion medium consisting of hydroxypropyl cellulose, Polysorbate 80, D‐mannitol, glucose and benzalkonium chloride were transferred into a vessel mixer of NP‐100 (THINKY CORPORATION) with yttrium‐stabilized zirconia milling bead. The pulverization was conducted to obtain nano‐crystalized regorafenib ophthalmic formulations with nano‐crystals at concentrations of 1.7 mg/mL (average particle size: 97.42 nm) and 2.1 mg/mL (average particle size: 233.8 nm).

Pazopanib eye‐drops at 4.9‐5.0 mg/mL was prepared by mixing pazopanib bulk powder and dissolving medium consisting of captisol, sodium dihydrogen phosphate and sodium chloride.

### 2.4 Pharmacokinetic study in rats

Regorafenib eye‐drops at 21.2 mg/mL or pazopanib eye‐drops at 4.9 mg/mL was administered as a single topical dose (10 μL/eye) to eyes in rats. Blood samples were collected from the inferior vena cava under anesthesia, and plasma samples were prepared by centrifugation of the blood samples. The both eyes (dosed eye and nondosed eye) were collected and washed by water, and then the eye tissues (the choroid/sclera and retina) were collected. Sample collections were performed at 0.5, 1.5, 4, 7, 24, and 96 hours (n = 2 at each time) after the ocular instillation of regorafenib eye‐drops and at 0.5, 1.5, 4, 24, 72, 120 and 168 hours (n = 2 at each time) after the ocular instillation of pazopanib eye‐drops.

A typical methods for sample preparation and determination of drug concentrations are shown herein. An aliquot of acetonitrile solution containing an internal standard was added to the plasma sample and mixed. After centrifugation, supernatant was mixed with 10 mmol/L ammonium acetate solution to prepare a sample for determination of drug concentration by liquid chromatography‐tandem mass spectrometry (LC/MS/MS). An aliquot of 50 vol% methanol was added to the eye tissues, and then homogenized by a beads shocker to prepare homogenate samples. An aliquot of the acetonitrile solution containing the internal standard was added to the homogenate samples and mixed. After centrifugation, supernatant was mixed with 10 mmol/L ammonium acetate solution to prepare samples for the determination of drug concentrations.

LC/MS/MS system consisted of ACQUITY UPLC system (Waters Corporation) and QTRAP6500 (AB SCIEX) was used for the determination of drug concentrations. The prepared samples were separated by ACQUITY UPLC peptide BEH C18 column (1.7 µm, 2.1 × 50 mm) (Waters Corporation, Milford, MA), with mobile phase A: 10 mmol/L ammonium acetate solution and mobile phase B: acetonitrile. After injection of the prepared samples to LC/MS/MS, the samples were eluted (0.2 mL/minutes) with 30% B followed 0.10 minutes isocratic period; 3.90 minutes linear gradient to 70% B; 0.01 minutes linear gradient to 30% B; and 1.49 minutes isocratic period. Multiple reaction monitoring was performed by using an atmospheric pressure chemical ionization probe, and monitor ion of regorafenib and pazopanib were 482.958/270.000 (m/z, Q1/Q3) and 438.184/356.900 (m/z, Q1/Q3), respectively.

The pharmacokinetic parameters of regorafenib and pazopanib in the eye tissues and plasma were calculated by average concentrations in the eye tissues and plasma. Maximum concentration (C_max_) and the time to reach C_max_ (t_max_) were obtained directly from the observed values. Log‐transformed concentrations were plotted against time. The elimination rate constant (k_e_) was estimated by linear least‐squares method using the last 2 or 3 observed data points. Apparent half‐life (t_1/2_) was obtained as ln2/k_e_. Area under the concentration vs time curve (AUC) from time 0 to the observed data point (AUC_0‐t_) was calculated using the linear trapezoidal method. AUC after the last data point was estimated by extrapolating with the k_e_ and added up with AUC_0‐t_ to calculate AUC from time 0 to infinity (AUC_0‐∞_).

### 2.5 Pharmacokinetic studies in rabbits and monkeys

In rabbits, regorafenib eye‐drops at 24.1 mg/mL (20 μL/eye) or pazopanib eye‐drops at 5.0 mg/mL (20 μL/eye) was administered as a single topical dose, and blood samples and eye tissue samples (the choroid/retina) were collected at 1.5 hours after ocular instillation of regorafenib eye‐drops (n = 3 for each sample) or pazopanib eye‐drops (n = 2 for each sample). In addition, nano‐crystalized regorafenib ophthalmic formulations with nano‐particles at concentrations of 1.7 mg/mL (average particle size: 97.42 nm) and 2.1 mg/mL (average particle size: 233.8 nm) were administered to rabbits and studied pharmacokinetics in the same manner described above.

In monkeys, regorafenib eye‐drops at 17.1 mg/mL (50 μL/eye) or pazopanib eye‐drops at 5.0 mg/mL (30 μL/eye) was administered as a single topical dose, and eye tissue samples (the cornea, iris/ciliary body, retina, and choroid, n = 2 for each sample) were collected at 4 hours after ocular instillation of regorafenib eye‐drops or pazopanib eye‐drops.

In the rabbit and monkey studies, sample preparation and determination of drug concentrations were performed in the same manner for the rat pharmacokinetic study.

### 2.6 Pharmacological study of regorafenib eye‐drops in laser‐induced rat CNV model

Pharmacological study in laser‐induced rat CNV model was conducted by referring to and optimizing the previous research.^27^ Animals with no ocular abnormalities were used for the study. The animals were arranged in ascending order of body weights and animals close to the median of the body weights were serially allocated into groups (13 animals per group).

#### 2.6.1 Induction of CNV

After the induction of mydriasis by instillation of Mydrin‐P ophthalmic Solution has been confirmed, the animals were anesthetized by intramuscular injection with a mixture solution of ketamine hydrochloride/xylazine hydrochloride (7/1, v/v). The ocular fundus of the eye of each animal was observed using a slit lamp (SL‐130; Carl Zeiss Meditec AG). Laser was irradiated on eight sites of the retina using a Multicolor Laser Photocoagulator (MC‐300; NIDEK Co., Ltd.) to induce CNV. The laser irradiation conditions were as follows; wavelength: 532 nm, spot size: 80 μm, irradiation time: 0.05 seconds, and laser output: 120 mW.

#### 2.6.2 Administration of test articles

Regorafenib eye‐drops and aflibercept were used as test articles. Just after the laser irradiation (the day of the laser irradiation was the start date of repeated ocular instillation), vehicle eye‐drops and regorafenib eye‐drops at 21.2 mg/mL were topically administered twice daily to the eyes induced CNV for 14 days. Just after the laser irradiation, aflibercept (200 μg/eye) was administered by intravitreal injection once.

#### 2.6.3 CNV evaluation

4 w/v% FITC‐dextran solution was administered into the tail vein of the animals under anesthesia by intramuscular injection of ketamine hydrochloride/xylazine hydrochloride (7/1, v/v). The animals were euthanized by an overdose of pentobarbital sodium under anesthesia. The eyes were collected and fixed in 4% paraformaldehyde‐phosphate buffer solution. Then, choroidal flat mounts were prepared by using a stereoscopic microscope (EZ‐4; Leica Microsystems).

Photographs of CNV sites were taken using a confocal microscope (ECLIPSE TE2000‐U; Nikon Instech Co., Ltd.). The areas of CNV sites were calculated using ImageJ Software (National Institutes of Health).

The CNV areas of the laser‐irradiated eight sites/eye, excluding data unsuitable for evaluation (eg, irradiation sites could not be confirmed; ill‐defined border; damaged sample), were used for analysis. The mean value of CNV areas was calculated for each animal with at least three irradiation sites available for analysis. The mean value was regarded as individual representative value.

#### 2.6.4 Statistical analysis

Dunnett test was performed between in the vehicle eye‐drops dosed group and the each test article dosed group. Stat Light #03 and Stat Light #04 (Yukms Co. Ltd., Kanagawa, Japan) were used these statistical analyses at a two‐sided significance level of 5%.

### 2.7 Pharmacological study of pazopanib eye‐drops in laser‐induced monkey CNV model

Pharmacological study in laser‐induced monkey CNV model was conducted by referring to and optimizing the previous researches.^28^, ^29^, ^30^ Animals were allocated to groups to minimize bias in the incidence and severity of CNV lesions and body weight between the groups.

#### 2.7.1 Induction of CNV

After the induction of mydriasis by instillation of Mydrin‐P ophthalmic solution has been confirmed, animals were anesthetized by intramuscular injection with a mixture solution of ketamine hydrochloride/xyladine (7/1, v/v). A fundus contact lens (Centralis Direct; Volk Optical Inc) was attached to the cornea with Scopisol solution and the position of the macula was confirmed. Laser was irradiated on the macula to induce CNV (8 sites per eye, 6 eyes per group). The laser irradiation conditions were as follows; wavelength: 532 nm, spot size: 80 μm, irradiation time: 0.1 seconds, and laser output: 1000 mW. The laser irradiation was conducted 21 days before the initiation of ocular instillation of test articles.

#### 2.7.2 Administration of test articles

Pazopanib eye‐drops and aflibercept were used as test articles. Repeated ocular instillation of vehicle eye‐drops or pazopanib eye‐drops at 5.0 mg/mL four times daily to the animals was initiated 22 days after the induction of CNV, and the repeated ocular instillation period was 35 days. Aflibercept (500 μg/eye) was administered by intravitreal injection to the animals once 22 days after the laser irradiation.

#### 2.7.3 Fluorescein fundus angiography and CNV grade evaluation

Fluorescein fundus angiography and CNV grade evaluation were conducted 1 day before the initiation of administration of test articles (Day‐1) and on Day8, Day15, Day22, Day29, and Day35 of the repeated ocular instillation of vehicle eye‐drops and pazopanib eye‐drops. In the aflibercept dosed group, fluorescein fundus angiography and CNV grade evaluation were conducted at the same time points in eye‐drops dosed groups.

Fluorescite Intravenous Injection 500 mg was administered into the cephalic vein under mydriasis and anesthesia. Photographs of fundus were taken with a fundus camera (VX‐10α; Kowa Co., Ltd., Aichi, Japan) approximately 1, 3, and 5 minutes after the administration of fluorescein. Each CNV lesion was graded as follows; Grade 1: no hyperfluorescence, Grade 2: hyperfluorescence without leakage, Grade 3: hyperfluorescence in the early (1 minute after administration of fluorescein) or midtransit (3 minutes after administration of fluorescein) images and late (5 minutes after administration of fluorescein) leakage, Grade 4: bright hyperfluorescence in the early or midtransit images and late leakage beyond the treated area in reference to the previous studies.^28^, ^29^, ^30^ The incidences of Grade 1, Grade 2, Grade 3 and Grade 4 lesions in the each eye (6 eyes per group) were calculated by the following equation, the incidence of grade lesion (%) = the number of grade lesions/ 8 × 100. The mean value of the incidences of Grade 4 lesions was calculated from 6 individual data in each group.

#### 2.7.4 Statistical analysis

Fisher's exact test was performed for the incidence of Grade 4 lesions between Day‐1 and Day 35 in each group. MiTOX System (Mitsui E&S Systems Research Inc (former company name; Mitsui Zosen Systems Research Inc) was used for the statistical analysis at a two‐sided significance level of 5%.

## 3 RESULT

### 3.1 Ocular pharmacokinetics of regorafenib and pazopanib in rats

**Figure 1:**
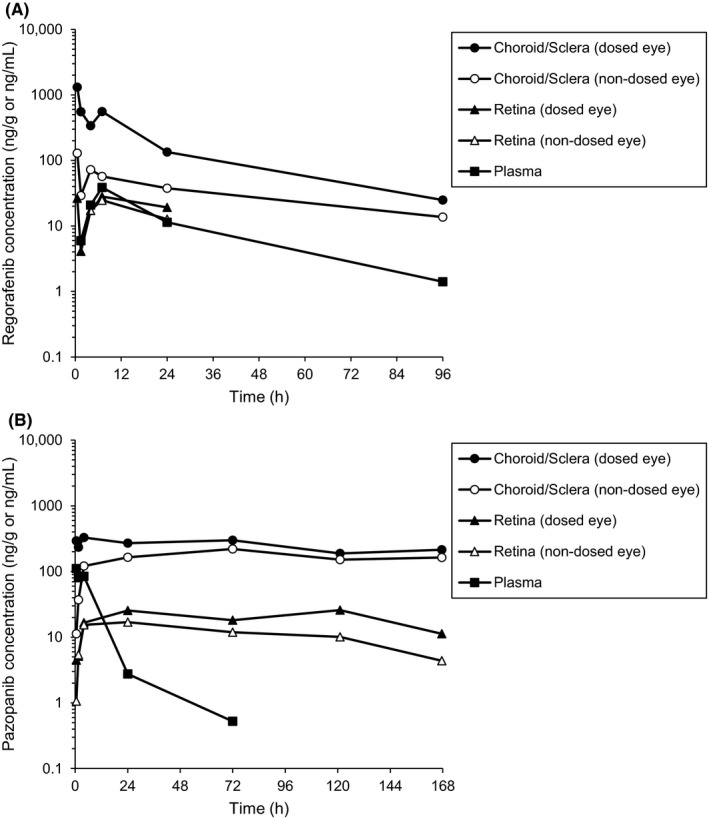
Ocular and plasma pharmacokinetics of regorafenib and pazopanib in rat. (A) Regorafenib concentrations in the choroid/sclera, retina, and plasma after a single ocular instillation of regorafenib eye‐drops at 21.2 mg/mL (10 μL/eye) to rats. (B) Pazopanib concentrations in the choroid/sclera, retina, and plasma after a single ocular instillation of pazopanib eye‐drops at 4.9 mg/mL (10 μL/eye) to rats. Each point represents the average of two animals. Units are nanograms per gram for the choroid/sclera and retina, and nanograms per milliliter for the plasma

Regorafenib and pazopanib were administered to one designated eye of rats, and the concentrations in the choroid/sclera, retina and plasma were measured (Figure 1). In addition, the ocular and plasma pharmacokinetic parameters were calculated (Table 1). The choroid/sclera concentrations of regorafenib (1310 ng/g) and pazopanib (293 ng/g) of the dosed eye at 0.5 hours after administration were much higher than those of the nondosed eye (regorafenib: 129 ng/g, pazopanib: 11.3 ng/g), suggesting efficient delivery of regorafenib and pazopanib from the instillation site. Since plasma concentrations of regorafenib and pazopanib were observed, regorafenib and pazopanib in the nondosed eyes were considered to come from systemic blood. Regorafenib and pazopanib concentrations in the retina were lower than those in the choroid/sclera and comparable between the dosed and nondosed eyes. This also suggested that regorafenib and pazopanib distributed into the retina mainly via system blood. The elimination half‐lives of regorafenib in the choroid/sclera (29.7 hours) and plasma (23.9 hours) were comparable. On the other hand, the elimination half‐lives of pazopanib in the choroid/sclera (245 hours) and retina (159 hours) of the dosed eye were much longer than that in the plasma (20.1 hours).

**Table 1:** Ocular and plasma pharmacokinetic parameters of regorafenib and pazopanib in the choroid/sclera, retina, and plasma of the dosed eye after a single ocular instillation of regorafenib eye‐drops at 21.2 mg/mL (10 μL/eye) and pazopanib eye‐drops at 4.9 mg/mL (10 μL/eye) to rats

Compound	Tissue	t_max_ (h)	C_max_ (ng/g or ng/mL)	AUC_0‐t_ (h × ng/g or h × ng/mL)	AUC_0‐∞_ (h × ng/g or h × ng/mL)	t_1/2_ (h)
Regorafenib	Choroid/Sclera	0.5	1310	15 300	16 400	29.7
	Retina	7	28.0	517	NC	NC
	Plasma	7	38.6	1010	1060	23.9
Pazopanib	Choroid/Sclera	4	330	42 100	112 000	245
	Retina	120	25.8	3450	6660	159
	Plasma	0.5	111	1280	1300	20.1

Note

Values are obtained or calculated by average tissue concentrations. Units are nanograms per gram for the choroid/sclera and retina, and nanograms per milliliter for the plasma

NC, Not calculable.

### 3.2 Species difference of ocular pharmacokinetics of regorafenib and pazopanib in rats, rabbits, and monkeys

Regorafenib and pazopanib concentrations in the posterior eye tissues were evaluated in rats, rabbits, and monkeys after a single ocular instillation (Table 2). Surprisingly, the regorafenib and pazopanib concentrations in the posterior eye tissues of the dosed eye were much lower in rabbits and monkeys compared to rats. Large species difference was observed in ocular pharmacokinetics of regorafenib and pazopanib after ocular instillation between animals.

**Table 2:** The regorafenib and pazopanib concentrations in the posterior eye tissues after a single ocular instillation of regorafenib eye‐drops and pazopanib eye‐drops to rats, rabbits, and monkeys. (A) Regorafenib concentrations in the posterior eye tissues of the dosed eye after a single ocular instillation of regorafenib eye‐drops to rats, rabbits, and monkeys. (B) Pazopanib concentrations in the posterior eye tissues of the dosed eye after a single ocular instillation of pazopanib eye‐drops to rats, rabbits, and monkeys. Values are the average of two animals

A				
Species	Rat		Rabbit	Monkey
Formulation conc. (mg/mL)	21.2		24.1	17.1
Dosed volume (mL)	0.01		0.02	0.05
Sampling time (h)	1.5	4	1.5	4

Tissue	Choroid/Sclera		Choroid/Sclera		Choroid/Retina		Choroid	
	Dosed eye	Nondosed eye	Dosed eye	Nondosed eye	Dosed eye	Nondosed eye	Dosed eye	Nondosed eye
Concentration (ng/g)	552	29.1	340	72.3	BLQ (<1)	BLQ (<1)	1.98	BLQ (<3)

B				
Species	Rat		Rabbit	Monkey
Formulation conc. (mg/mL)	4.9		5.0	5.0
Dosed volume (mL)	0.01		0.02	0.03
Sampling time (h)	1.5	4	1.5	4

Tissue	Choroid/Sclera		Choroid/Sclera		Choroid/Retina		Choroid	
	Dosed eye	Nondosed eye	Dosed eye	Nondosed eye	Dosed eye	Nondosed eye	Dosed eye	Nondosed eye
Concentration (ng/g)	234	37.0	330	121	14.5	5.73	18.3	19.2

BLQ, Below the lower limit of quantification.

### 3.3 Ocular distribution of regorafenib and pazopanib in monkeys

More detailed ocular distribution including the anterior eye tissues were evaluated for regorafenib and pazopanib after a single ocular instillation to monkeys (Table 3). Compared to the posterior eye segments, higher concentrations of regorafenib were observed in the cornea and iris/ciliary body in the dosed eye while the concentrations in the nondosed eye were below the lower limit of quantification in both the anterior and posterior segments. Similarly, pazopanib concentrations in the anterior eye tissues were higher than those in the posterior eye tissues. Pazopanib concentrations in the iris/ciliary body, retina, and choroid were similar in both dosed and nondosed eyes, suggesting that the major distribution is via systemic blood.

**Table 3:** Ocular distribution of regorafenib and pazopanib in monkeys. The regorafenib and pazopanib concentrations in the eye tissues of the dosed eyes and nondosed eyes after a single ocular instillation of regorafenib eye‐drops at 17.1 mg/mL (50 μL/eye) and pazopanib eye‐drops at 5.0 mg/mL (30 μL/eye) to monkeys. Values are the average of two animals

Tissue	Regorafenib concentration (ng/g)		Pazopanib concentration (ng/g)	
	Dosed eye	Nondosed eye	Dosed eye	Nondosed eye
Cornea	350	BLQ (<3)	83.8	BLQ (<1)
Iris/Ciliary body	49.4	BLQ (<3)	10.1	7.82
Retina	BLQ (<3)	BLQ (<3)	3.23	2.77
Choroid	1.98	BLQ (<3)	18.3	19.2

BLQ, Below the lower limit of quantification.

### 3.4 Laser‐induced rat CNV model

**Figure 2:**
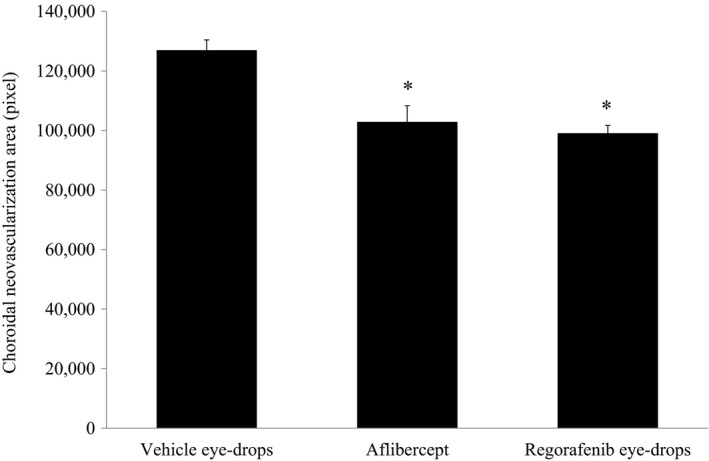
CNV area in laser induced rat CNV model. CNV was induced by laser irradiation (eight sites per eye, 12‐13 eyes per group). Just after the laser irradiation, vehicle eye‐drops and regorafenib eye‐drops at 21.2 mg/mL were topically administered twice daily to the animals for 14 days. Aflibercept (200 μg/eye) was also administered by intravitreal injection to the animals once just after the laser irradiation. After the administration of vehicle eye‐drops and test articles, the areas of CNV were evaluated. Mean value and standard error were calculated from 12‐13 animals in each group. Statistical analysis based on analysis of variance with Dunnett's test was performed; *P < .05 (all comparisons to vehicle eye‐drops dosed group)

The inhibitory effects of regorafenib on laser‐induced CNV were evaluated in rats after repeated ocular instillations (21.2 mg/mL, twice daily, 14 days). Aflibercept was also tested as a positive control by a single intravitreal injection (200 μg/eye). In the aflibercept dosed group, CNV was significantly suppressed (*P* < .05). In the regorafenib eye‐drops dosed group, significant reduction in CNV area was also observed (*P* < .05), and the efficacy was comparable to that of aflibercept (Figure 2).

### 3.5 Laser‐induced monkey CNV model

**Figure 3:**
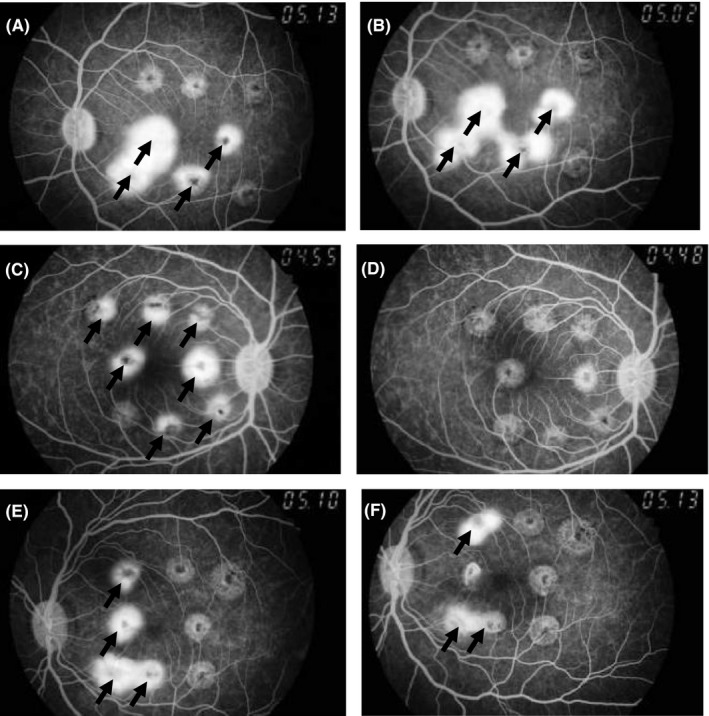
Fluorescein fundus angiograms in laser induced monkey CNV model. Photographs of fundus were taken approximately 5 minutes after the administration of fluorescein. Typical fluorescein fundus angiograms of the same animal on Day‐1 (A) and Day35 (B) in the vehicle eye‐drops dosed group. Typical fluorescein fundus angiograms of the same animal on Day‐1 (C) and Day35 (D) in the aflibercept dosed group (500 μg/eye). Typical fluorescein fundus angiograms of the same animal on Day‐1 (E) and Day35 (F) in the pazopanib eye‐drops dosed group (5.0 mg/mL, four times daily, 35 days). Arrow represents Grade 4 lesion

The inhibitory effects of pazopanib (5.0 mg/mL, 4 times daily, 35 days) on laser‐induced CNV were evaluated in monkeys after repeated ocular instillations (Figure 3 and Table 4). Aflibercept (500 μg/eye) was also tested as a positive control by a single intravitreal injection. The incidence of Grade 4 lesions was used as an index for the inhibitory effects of both compounds. The incidences of Grade 4 lesions in vehicle dosed group at predosing (Day‐1) and on Day35 were 31.3% and 20.8%, respectively, and no significant difference was observed between the two days. In aflibercept dosed group, the incidence of Grade 4 lesions on Day‐1 was 43.8%, and that significantly reduced to 4.2% on Day8. The significant efficacy had been maintained until Day35 (*P* < .0001). On the other hand, the incidences of Grade 4 lesions on Day‐1 and Day35 in the pazopanib dosed group were 50.0% and 43.8%, respectively, with no significant difference between Day‐1 and Day35.

**Table 4:** Percentage of Grade 4 lesion in laser induced monkey CNV model. CNV was induced by laser irradiation (8 sites per eye, 6 eyes per group). The laser irradiation was conducted 21 days before the initiation of administration of test articles, and then CNV was induced. Vehicle eye‐drops or pazopanib eye‐drops at 5.0 mg/mL was topically administered four times daily to the animals for 35 days. Aflibercept (500 μg/eye) was also administered by intravitreal injection once. CNV grade evaluation was conducted 1 day before the initiation of administration of test articles (Day‐1) and on Day8, Day15, Day22, Day29, and Day35 of the repeated ocular instillation of vehicle eye‐drops and pazopanib eye‐drops. In the aflibercept dosed group, CNV grade evaluation was also conducted at the same time points to eye‐drops dosed groups. Statistical analysis based on Fisher's exact test was performed for the incidences of Grade 4 lesions between Day‐1 and Day 35

Group		Percentage of Grade 4 lesion (%)					
		Day‐1	Day 8	Day 15	Day 22	Day 29	Day 35
Vehicle eye‐drops	Mean	31.3	22.9	18.8	20.8	20.8	20.8
	SE	13.2	13.1	12.0	11.5	11.5	11.5
Pazopanib eye‐drops	Mean	50.0	47.9	43.8	45.8	43.8	43.8
	SE	10.7	15.9	15.7	15.0	15.1	15.1
Aflibercept	Mean	43.8	4.2	4.2	4.2	4.2	4.2*
	SE	11.5	4.2	4.2	4.2	4.2	4.2

SE, standard error.

* *P* < .0001

### 3.6 Nano‐crystal to increase drug delivery to the posterior eye tissues

In order to evaluate the impact of nano‐crystalization on the ocular delivery to the posterior eye segments, regorafenib formulations were prepared with multiple mean particle sizes: 97.42, 233.8, and 6400 nm and used in the pharmacokinetic study in rabbits (Table 5). After a single ocular instillation (20 μL/eye) of these formulations, regorafenib concentrations in the choroid/retina were compared at 1.5 hours postdose. No regorafenib concentration was detected in the choroid/retina for the micro‐sized (6400 nm) formulation. On the other hand, regorafenib concentrations were detected with nano‐sized (97.42 and 233.8 nm) formulations.

**Table 5:** The regorafenib concentrations in the choroid/retina after a single ocular instillation of regorafenib ophthalmic formulations to rabbits. Values are the average and standard deviation of three animals

Formulation conc. (mg/mL)	1.7	2.1	24.1
Average particle size (nm)	97.42	233.8	6400
Choroid/retina conc. (ng/g)	3.73 ± 0.56	1.24 ± 1.35	BLQ (<1)

NC, Not calculable.

## 4 DISCUSSION

In this study, we evaluated interspecies differences in ocular pharmacokinetics after ocular instillation of regorafenib and pazopanib in rats, rabbits, and monkeys. In rats, regorafenib and pazopanib showed efficient ocular delivery to the choroid/sclera of the dosed eye. Regorafenib and pazopanib were also detected in the choroid/sclera of the nondosed eye although the concentrations in the nondosed eye were lower than those in the dosed eye. This may be explained by the distribution via systemic blood since regorafenib and pazopanib were also detected in the plasma. It was reported that, instilled dose solution is rapidly washed away from the precorneal area by lacrimal fluid and then is drained through the nasolacrimal duct. Afterwards, the swollen dose solution can undergo intestinal absorption.^31^, ^32^, ^33^ Regorafenib and pazopanib detected in the plasma in rats may have come from intestinal absorption of the dose solution. In addition, the drug absorption in the eye from instillation site should be accounted by subtracting the concentration in the nondosed eye from that in the dosed eye.^34^ Regorafenib and pazopanib concentrations in the retina were similar between the dosed and nondosed eyes in rats, suggesting that they are mainly distributed from systemic blood. The elimination half‐lives of regorafenib in the choroid/sclera and plasma were comparable. On the other hand, the elimination half‐lives of pazopanib in the choroid/sclera and retina were much longer than that in the plasma. Pazopanib strongly binds to melanin and that may contribute the long retention of pazopanib in the melanin containing tissues, such as the choroid/sclera and retina.^35^

For both regorafenib and pazomanib, the choroid/sclera concentrations after ocular instillation in rats were high enough compared to the inhibitory concentrations against VEGFR‐2 until 96‐168 hours (IC_50_; Regorafenib: 10‐40 nmol/L, Pazopanib: 17‐42 nmol/L).^36^, ^37^ Reflecting this, regorafenib showed significant reduction in CNV in rat model. The efficacy was comparable to that of aflibercept after intravitreal injection. Previous study demonstrated that pazopanib also reduced angiogenesis and vascular hyperpermeability after ocular instillation in rats.^22^

However, regorafenib and pazopanib concentrations in the choroid and retina after ocular instillations were much lower in rabbits and monkeys than those in rats. The concentrations of these drugs in the posterior eye tissues in rabbits and monkeys seemed to be insufficient to suppress angiogenesis and vascular hyperpermeability based on VEGFR inhibition. The concentrations of regorafenib and pazopanib in the anterior eye tissues were higher than those in the posterior eye tissues, suggesting limited delivery of the compounds from the anterior to posterior sites in monkeys. Pazopanib concentrations in the choroid and retina of the dosed eye and nondosed eye were comparable in monkeys, so it is possible that most of pazopanib in the posterior eye tissues were transferred from the systemic blood, not from the instillation site. Although we evaluated the efficacy of pazopanib in the monkey CNV model, no clear efficacies were observed after repeated ocular instillation of pazopanib, suggesting that it is possible that insufficient exposure was the cause of the lack of efficacy in monkeys. In other study, ocular instillation of pazopanib failed to suppress VEGF‐induced fluorescein leakage in the posterior eye tissues in rabbits.^38^ As the results showed above, there is a significant interspecies difference in drug delivery to the posterior eye tissues and pharmacological activities after ocular instillation of regorafenib and pazopanib among rat, rabbit, and monkey.

To take into consideration the relationship between pharmacokinetics and pharmaceutical activities of a compound, tissue binding of the compound should be considered. However, it is difficult to evaluate unbound fractions of the compound in the specific eye tissues, since the choroid and retina have complex compositions including melanin. So it is hard to determine specific compositions and unbound fractions related to drug efficacy. Collecting enough tissues for an evaluation of unbound fraction is also not easy, particularly from small animals including rats. In addition, to consider interspecies differences in drug delivery to the posterior eye tissues after ocular instillation of a compound, allometry of eye size and dosage of the compound are important. There is a large interspecies difference in eye sizes (the vitreous volume; rats: 0.013‐0.054 mL, rabbits: 1.5‐1.8 mL, monkeys: 1.8‐2.0 mL, human: 4 mL).^39^ For the evaluation of drug transfer efficiency, instillation dose level should be arranged by adjusting a drug concentration in a dosing formulation and an instillation volume in each animal. In this study, we found out that there is a significant interspecies differences in total drug concentrations in the posterior eye tissues and pharmacological activities after ocular instillation of regorafenib and pazopanib among rat, rabbit, and monkey, though, further studies are needed in order to consider ocular pharmacokinetics and pharmaceutical activities in the multiple animals and also in human.

Nano‐crystals, also regarded as ‘nano‐suspensions’, are drug crystals with nanometer‐size particles and can be applied to insoluble drugs.^25^ Conventional ophthalmic formulations result in less than 5% bioavailability of dosed drugs to the eye tissues because of the ocular structural barriers and rapid draining by lacrimal fluid.^33^ Nano‐crystal technology can increase the adhesiveness to tissue surface, the dissolution rate, and apparent solubility of poorly soluble drugs.^23^, ^24^, ^25^, ^26^ Therefore, it is expected that nano‐crystalization can improve drug delivery to the posterior segment of eye after ocular instillation of insoluble drugs, such as regorafenib. On the other hand, pazopanib is a soluble drug, so that is the reason why we judged that it was difficult to apply nano‐crystal technology to pazopanib. We developed nano‐crystallized regorafenib ophthalmic formulations with particle sizes ranging from around 100 nm to 230 nm, and studied to see if nano‐crystallization is useful to increase the drug delivery of regorafenib to the posterior eye tissues. Although no exposure of regorafenib in the choroid/retina was observed with micro‐crystal suspension (average particle size: 6.4 μm) at a higher concentration of 24.13 mg/mL in rabbits, detectable regorafenib concentrations in the choroid/retina were observed with nano‐crystalized formulations (average particle size: 97.42‐233.8 nm) at lower concentrations of 1.72‐2.06 mg/mL, suggesting that nano‐crystallization can improve the drug delivery of regorafenib to the posterior eye tissues. Since the experiment was conducted at the limited time point, further studies with enough time points to evaluate detailed ocular harmacokinetics would be required to accurately consider the contribution of nano‐crystallization to drug transfer.

In conclusion, significant species differences were observed in drug delivery to the posterior eye tissues after ocular instillation of regorafenib and pazopanib among rat, rabbit, and monkey, and it is possible that the insufficient exposure of these drugs in the posterior eye tissues was one of causes of the lack of efficacies in rabbits, monkeys, and also patients with neovascular AMD. Nano‐crystal technology seems to be useful to increase drug delivery of regorafenib to the posterior segment of eye. Further studies are needed in order to consider causes of the lack of efficacy of these drugs in clinical and develop nano‐crystalized regorafenib ophthalmic formulation which expresses enough efficacies in patients with neovascular AMD.

## DISCLOSURE

We state that there is no conflict of interest in our manuscript.

## AUTHOR CONTRIBUTIONS

Shinya Horita: Constructed research design. Conducted the experiments. Performed data analysis. Wrote the manuscript.

Miwa Watanabe and Hiroki Haniuda: Conducted the experiments. Contributed to the writing of the manuscript.

Mai Katagiri: Conducted the experiments.

Hiroaki Nakamura and Tomoyuki Nakazato: Contributed to the writing of the manuscript.

Yoshiyuki Kagawa: Participated in research design. Contributed to the writing of the manuscript.
